# An Unusual Case of Schwann Cell Hamartoma in Colon

**DOI:** 10.7759/cureus.39301

**Published:** 2023-05-21

**Authors:** Sanna Salam, Hazem Abosheaishaa, Muhammad Haseeb ul Rasool, Nimra Qasim, Ghullamullah Shahzad

**Affiliations:** 1 Medicine, Queens Hospital Center, New York City, USA; 2 Internal Medicine, Icahn School of Medicine at Mount Sinai, Queens Hospital Center, New York City, USA; 3 Internal Medicine/Gastroenterology, Cairo University, Cairo, EGY; 4 Medicine, Icahn School of Medicine at Mount Sinai, Queens Hospital Center, New York City, USA; 5 Gastroenterology, Liaquat University of Medical and Health Sciences, Jamshoro, PAK; 6 Gastroenterology, NYC Health + Hospitals/Queens, New York City, USA; 7 Gastroenterology, Icahn School of Medicine at Mount Sinai, New York City, USA

**Keywords:** schwann cell, colonoscopy, bleeding per rectum, cancer colon, polyp

## Abstract

Schwann cell tumors are benign tumors originating from Schwann cells of the peripheral nervous system and are extremely rare in the gastrointestinal system. They usually originate in the colon or rectum but can also occur in the esophagus and small intestine. Their occurrence is rare in GI tract and mainly in the sigmoid colon. Schwann cell tumors have no association with any familial cancer syndromes. We present a 65-year-old female patient who underwent routine colon cancer screening. In addition to open mouth diverticulosis, she was found to have a 3 mm polyp, which was diagnosed as a Schwann cell hamartoma after a biopsy. This study aimed to present this rarely reported case in the literature as an example of a tumor that should be included in the differential diagnosis when considering submucosal colonic lesions. Though the reported reoccurrence rate is low, this case highlights the lack of published guidelines regarding appropriate follow-up surveillance periods.

## Introduction

Colonic mucosal Schwann cell hamartoma (MSCH) are relatively rare and benign tumors in the gastroenterology system. They are so named because they develop from the perineural Schwann cells and are thus classified as nonepithelial tumors. They are composed of lymphoid cuffing, atypical nuclear cells, and spindle cells. Histologically, these cells are polygonal with numerous eosinophilic granules and spindle cells which are usually positive for S-100. To the best of our knowledge, only 35 cases have been reported to date, all of which were accidentally found during colonoscopy screenings. This study aimed to raise awareness about a rare form of MSCH.

## Case presentation

A 65-year-old female patient with a medical history of hypertension, hyperlipidemia, gastroesophageal reflux disease, and diverticulosis presented for a routine colonoscopy screening for colorectal cancers. She had a significant personal and family history of malignancies, including two different triple-negative stage-IIB invasive mammary carcinomas with squamous features and a stage-IA invasive mammary ductal carcinoma, for which she had undergone modified radical mastectomy, adjuvant chemotherapy, and radiotherapy. Her family history was significant for prostate and pancreatic cancers in first-degree relatives. A screening colonoscopy performed five years ago revealed a significantly scattered diverticulum in the sigmoid colon with no evidence of malignancies.

**Figure 1:**
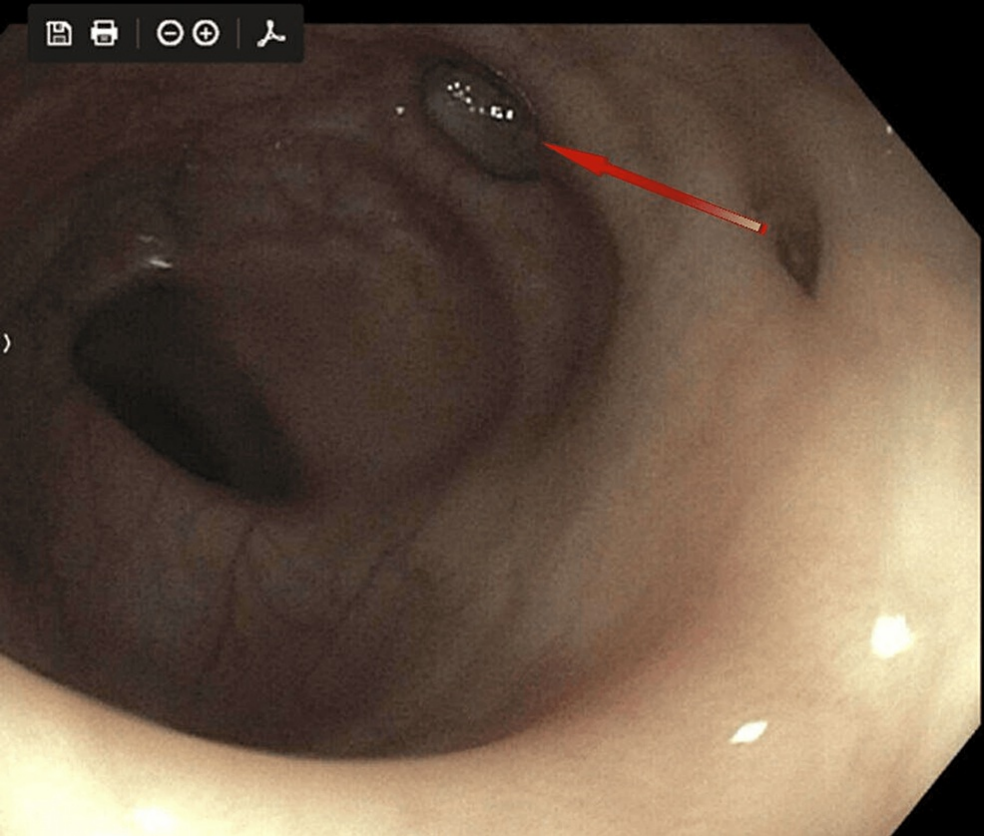
Colonoscopic finding of Schwann cell hamartoma.

This screening colonoscopy revealed multiple medium-mouthed diverticula in the sigmoid, transverse, and ascending colon. A 3 mm sessile polyp was identified and removed from the sigmoid colon using cold biopsy forceps (Figure 1).

**Figure 2:**
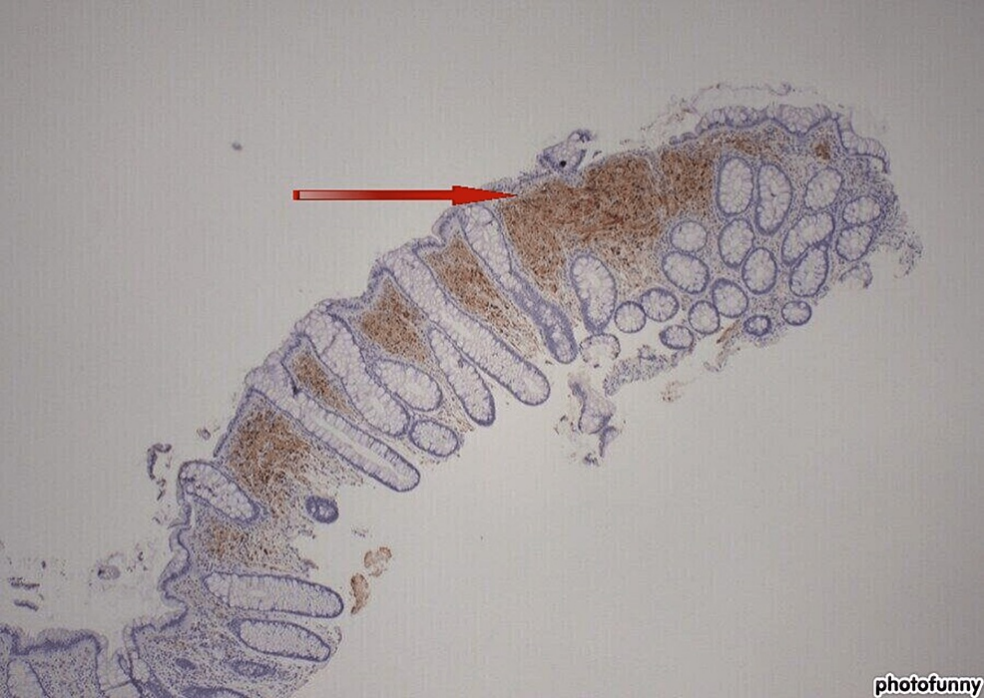
Microscopic finding of S-100 protein.

Histology of the polyp revealed sigmoid colonic mucosa with spindle cell proliferation, consistent with MSCH. The spindle cells tested positive for S-100, which is a marker commonly found in Schwann cells. However, the spindle cells tested negative for other markers, such as cluster of differentiation (CD)34, CD117, spinal muscle atrophy (SMA), desmin, and anti-mitochondrial antibody (AMA), which ruled out other types of tumors (Figure 2). After obtaining the results, computerized tomography (CT) was performed on the chest, abdomen, and pelvis, which did not reveal any other malignancy. She is currently being followed up on in the gastrointestinal and oncological clinic with a plan to perform a screening colonoscopy again in one year.

## Discussion

Compared to other types of gastrointestinal polyps, hamartomatous polyps are relatively rare; however, they are the most prevalent type of gastrointestinal polyps in children. Common symptoms include rectal bleeding, abdominal pain, constipation, anemia, and small bowel obstruction [1]. Colonic MSCH is very rare and usually discovered during routine colon screening as small polypoid intraluminal lesions, often with mucosal ulceration [2]. Gastrointestinal schwannomas differ from soft tissue schwannomas in several ways, including the presence of a reactive lymphoid peripheral cuff, the absence of encapsulation, and degenerative changes [3].

In 2009, Gibson and Hornick reported 26 neural colorectal polyps distinct from neurofibromas and mucosal neuromas [4]. Similarly, in 2011, Rocco et al. presented a case of a tiny colonic polyp with intramucosal scattered spindle cell growth, a benign cytological appearance, strong dispersed immunoreactivity for S-100 protein, and a pure Schwann cell phenotype [5]. Sagami et al. reported a case of MSCH in 2012, which was diagnosed after the presentation of anemia and a positive fecal occult blood test [6]. The literature review endorsed some MSCH polyps that have been detected in the colon during an incidental screening colonoscopy and confirmed by histopathological features with the presence of pure Schwann cell proliferation in the lamina propria and show diffuse immunoreactivity for the S-100 protein [7-11]. Moreover, in 2013, Neis et al. reported a case of MSCH in an ulcerative colitis patient with primary sclerosing cholangitis during routine screening [12].

Other than the colon, MSCH has been reported in the gastroesophageal junction by Li et al., who reported a series of six cases that presented as irregular Z lines and erosive lesions seen at the gastroesophageal junction [13]. Histopathology confirmed the same histopathological features as those in colonic MSCH. Additionally, Oguntuyo et al. [14] reported a case of MSCH in the duodenum, and Ismael et al. reported another case of MSCH in the gallbladder in a patient who presented with abdominal pain, common bile duct stones, gallbladder thickening, marked biliary dilatation, and pancreatic ductal dilatation [15].

Mucosal Schwann cell hamartoma is a very infrequent tumor. No relation has been documented to date between MSCH and neurofibromatosis, and no cases of malignant transformation have been confirmed. However, its clinical importance has yet to be determined. As a result, patients with MSCH require close and ongoing monitoring [7].

## Conclusions

Schwann cell hamartomata’s polyps are rare benign lesions that might implicate colon malignancy or aberrancy. It has not yet been reported in the sigmoid colon, as in this case. Given its rarity, awareness of this lesion with more studies is required for diagnosis, long-term screening, and follow-up guidelines.
